# Premature ventricular complexes and migraine: insights from Holter monitoring during pain-free intervals

**DOI:** 10.1055/s-0045-1813242

**Published:** 2025-12-08

**Authors:** Serhat Kesriklioglu, Muhammed Fatih Kaleli, Yakup Alsancak, Aysenur Ince, Mustafa Altas

**Affiliations:** 1 Necmettin Erbakan University, Faculty of Medicine, Department of Cardiology, Konya, Turkey.; 2 Kelkit State Hospital, Department of Neurology, Gumushane, Turkey.; 3 Necmettin Erbakan University, Faculty of Medicine, Department of Neurology, Konya, Turkey.

**Keywords:** Migraine Disorders, Ventricular Premature Complexes, Autonomic Nervous System Diseases, Electrocardiography, Ambulatory, Tachycardia, Sinus

## Abstract

**Background:**

Migraine is a common neurological disorder associated with an increased risk of cardiovascular conditions, including arrhythmias. Although autonomic dysfunction is considered a key mechanism linking migraine and cardiac abnormalities, its impact during pain-free intervals remains unclear.

**Objective:**

To investigate the association between migraine and cardiac arrhythmias, focusing on the prevalence of premature ventricular contractions (PVCs) and autonomic dysfunction during pain-free periods.

**Methods:**

A total of 50 migraine patients and 51 age- and sex-matched healthy controls were enrolled in the present observational, cross-sectional study. All participants underwent 24-hour Holter monitoring, standard electrocardiographic and echocardiographic evaluation. The frequencies of PVCs, PR interval, QT interval (QTc), heart rate variability, and sinus tachycardia prevalence were compared between groups. Migraine-related disability was assessed using the Migraine Disability Assessment Scale (MIDAS) and the Headache Impact Test-6 (HIT-6).

**Results:**

At least 1 PVC was detected in 40% of migraine patients, although no significant difference was observed compared with controls ( *p* > 0.05). However, migraine patients showed a significantly higher prevalence of sinus tachycardia ( *p* < 0.05). Additionally, a positive correlation was found between PVC burden and MIDAS scores, suggesting a potential link between migraine severity and arrhythmia risk. No significant differences were observed in QTc intervals or other major arrhythmic parameters.

**Conclusion:**

Migraine patients may exhibit increased sinus tachycardia and subtle electrocardiographic changes even during pain-free intervals, possibly reflecting underlying autonomic dysfunction.

## INTRODUCTION

Migraine is a prevalent and disabling neurological disorder, affecting > 1 billion people worldwide, with a particularly high prevalence among women < 50 years old. It is characterized by recurrent episodes of severe, unilateral headache often accompanied by autonomic symptoms such as nausea, vomiting, photophobia and phonophobia, which can markedly impair daily functioning. 1 2 3 4 The pathophysiology of migraine is complex and multifactorial, involving genetic, behavioral, and environmental factors. These contribute to altered sensory processing in the brain, leading to increased sensory susceptibility. Key mechanisms include cortical spreading depolarization, neuroinflammatory responses, and the release of neuropeptides, such as calcitonin gene-related peptide (CGRP), which collectively activate the trigeminocervical complex. 5 6

Beyond its neurological impact, migraine has been associated with an increased risk of cardiocerebrovascular disorders, including stroke, coronary artery disease (CAD), atrial fibrillation (AF), patent foramen ovale (PFO), and atrial septal defects (ASD). The mechanisms underlying these associations may involve endothelial dysfunction, inflammation, genetic predisposition, and vascular risk factors. 1 7 8 9 Autonomic nervous system dysregulation is also thought to play a significant role, with sympathetic–parasympathetic imbalance potentially contributing to both migraine symptoms and cardiovascular abnormalities. 10 11 Notably, migraine attacks have been associated with ventricular arrhythmias, such as premature ventricular contractions (PVCs), and alterations in electrocardiographic (ECG) parameters, including prolonged QT intervals (QTcs), supporting a role for autonomic dysregulation during attacks. 7 12 13

Premature ventricular contractions, defined as early ventricular depolarizations, are the most common asymptomatic ventricular arrhythmias detected on electrocardiography. 14 While they are often associated with structural heart conditions, they can also occur in otherwise healthy individuals. 15 16 Prior research suggests that ∼ 4% of people with normal coronary and echocardiographic findings have > 100 PVCs per day. 15

Most prior studies have focused on arrhythmias during acute migraine attacks. 7 12 13 In contrast, our study aims to explore the prevalence of PVCs and other arrhythmias during pain-free intervals between migraine episodes. This distinction is critical to determining whether autonomic imbalance persists beyond the acute phase of migraine and contributes to arrhythmia risk in the absence of active headache symptoms, or whether attack-free intervals are comparable to those of healthy individuals. By addressing this gap, we aim to provide new insights into the long-term cardiovascular implications of migraine.

## METHODS

The present study was designed as a single-center, observational, and cross-sectional analysis conducted to evaluate the association between migraine and cardiac arrhythmias, particularly focusing on the prevalence of PVCs during the nonattack period in migraine patients. Patients experiencing migraine who presented at a neurology outpatient clinic of a tertiary university hospital between April 15, 2021 and September 15, 2023 and healthy volunteers were included in the study. Ethical approval was obtained from the institution's ethics committee (decision number 4,448).

### Selection of participants and data collection

Migraine patients were recruited from a neurology outpatient clinic and diagnosed based on the International Classification of Headache Disorders (ICHD-3) criteria. The study included individuals aged between 18 and 65 years old with a confirmed diagnosis of episodic migraine. Patients with migraine diagnosed by a neurologist, either with aura (MWA) or without aura (MWoA), were included. The control group was composed of healthy volunteers who were matched to the migraine patients by age and sex. These individuals were selected among people who presented to the cardiology outpatient clinic solely for routine screening purposes. None of the controls were first-degree relatives of the patients or had a history of migraine. The participants were instructed to maintain their usual daily activities during the 24-hour Holter monitoring period, without any restrictions on physical activity.

The sample size was not based on formal power calculation. Instead, it was determined by the number of eligible patients and control participants available during the study period (between April 2021 and September 2023). This approach was deemed appropriate given the cross-sectional and exploratory nature of the study.

Exclusion criteria included structural heart disease, known arrhythmias, use of β-blockers, nondihydropyridine calcium channel blockers (verapamil/diltiazem), or antiarrhythmic drugs; heavy caffeine consumption (> 6,000 mg/day); stimulant use; baseline ECG showing tachycardia (> 100 beats per minute [bpm]) or bradycardia (< 60 bpm); pregnancy; or lack of informed consent.

### Measurements of electrocardiogram parameters

A 12-lead ECG was obtained with a paper speed of 10 mm/mV and 25 mm/s. All measurements were performed manually by a cardiologist who was blinded to the clinical laboratory and demographic information of the patients. The PR interval was measured from the onset of the P wave to the beginning of the QRS complex. The QT interval, expressed in milliseconds (ms), was measured from the start of the Q wave to the point where the T wave returns to the isoelectric line. The corrected QTc was calculated using Bazett's formula (QT/√R-R) to adjust for heart rate. The QTc for each lead was determined as the average value of three consecutive beats.

### Conventional echocardiography

Echocardiographic assessments, including 2D, M-mode, pulsed Doppler, and pulsed tissue Doppler imaging (TDI), were conducted using an echocardiography machine (VIVID S-5, General Electric Medical System Vingmed Ultrasound AS) with a 3.6-MHz transducer. Left ventricular (LV) volumes and ejection fraction (LVEF) were determined using the modified Simpson method from both apical four- and two-chamber views. Mitral inflow velocities were measured via pulse-wave Doppler with the sample volume positioned at the tip of the mitral leaflets from the apical four-chamber view. The diastolic peak early (E) and peak late transmitral flow velocities (A) were calculated as the average of three cardiac cycles.

### Measurements of 24-hour rhythm Holter parameters

A 24-hour rhythm Holter monitor (Spacelabs Healthcare) was used to record the following parameters: maximum heart rate (max HR, bpm), mean heart rate (mean HR, bpm), minimum heart rate (min HR, bpm), PVC count, supraventricular ectopy (SVE) count, and additional Holter findings (sinus tachycardia, sinus bradycardia, etc).

### Assessment of migraine parameters

The functional status of the patients over the past 3 months was evaluated using the Migraine Disability Assessment (MIDAS) questionnaire, which quantifies the level of disability caused by migraines in terms of missed work, social, and home activities. The Headache Impact Test (HIT-6) scores were also used to assess the impact of migraines on the patient's daily functioning and quality of life. Additionally, the severity of headaches during the attack period was assessed using the visual analogue scale (VAS), which allows patients to rate the intensity of their pain on a scale from 0 (no pain) to 10 (worst possible pain). These tools provided a comprehensive assessment of the migraine-related burden experienced by each patient.

### Statistical analysis

The data obtained from the study were analyzed using Statistical Package for Social Sciences version 25.0 (SPSS Inc.). In descriptive analyses, frequency data were presented as number ( *n* ) and percentage (%), while numerical data were presented as mean ± standard deviation (SD) and median (interquartile range [IQR]). For comparison of categorical variables, the Chi-squared (χ ^2^ ) test, the Fisher's Exact test, and the Fisher-Freeman-Halton test were applied. The normality of numerical data distribution was examined using the Kolmogorov-Smirnov test. The distribution of normally distributed numerical data in independent groups was analyzed using the Independent Samples *t* -test, while the distribution of non-normally distributed data was evaluated using the Mann-Whitney U-test. The distribution of numerical variables across more than two independent groups was analyzed using the Kruskal-Wallis test. The relationship between non-normally distributed numerical data was examined using Spearman's Correlation analysis. The correlation coefficients were interpreted as follows: r = 0.05 to 0.30 as weak correlation, r = 0.30 to 0.40 as low-moderate correlation, r = 0.40 to 0.60 as moderate correlation, r = 0.60 to 0.70 as good correlation, r = 0.70 to 0.75 as very good correlation, and r = 0.75 to 1.00 as excellent correlation.

Statistical significance was evaluated at the 95% confidence interval (CI) with a significance level of *p* < 0.05.

## RESULTS

The present study, conducted at Necmettin Erbakan University Faculty of Medicine Hospital, included 50 patients with migraine and 51 healthy participants. The distribution of data in both the patient and control groups is presented in Table 1 . A total of 90.0% ( *n* = 45) of the patients were female, compared with 76.5% ( *n* = 39) in the control group. The mean age of the patient group was 39.26 ± 11.90 years old. There were no statistically significant differences between the groups in terms of gender, age, or comorbidity distribution ( *p* > 0.05). The median duration of migraine diagnosis among patients was 36.0 months (IQR: 16.0–60.0 months) ( Table 1 ).

**Table 1:** Characteristics of the patient and control groups

Variable		Control ( *n* = 51)	Migraine patients ( *n* = 50)	*p-value*
Gender (female); *n* (%)		39 (76.5)	45 (90.0)	0.069 *
Age (years old)		35.90 ± 10.46	39.26 ± 11.90	0.135 **
DM; *n* (%)		1 (2.0)	5 (10.0)	0.112 ***
HT; *n* (%)		5 (9.8)	7 (14.0)	0.515 *
Comorbidity; *n* (%)		8 (15.7)	7 (14.0)	0.812 *
**Disease-related parameters in migraine patients**	Duration of diagnosis (months)		36.0 (16.0–60.0)	
	HIT-6		46.0 (42.0–55.2)	
	MIDAS		9.5 (7.0–16.2)	
	VAS		7.0 (7.0–8.0)	
	Aura, *n* (%)		6 (12.0)	
	Photophobia-Phonophobia, *n* (%)		36 (72.0)	
**Migraine prophylaxis**	None		23 (46.0)	
	Magnesium		8 (16.0)	
	Propranolol		1 (2.0)	
	Flunarizine		8 (16.0)	
	Valproate		1 (2.0)	
	Amitriptyline		9 (18.0)	

Abbreviations: DM, diabetes mellitus; HT, hypertension; HIT-6, Headache Impact Test-6; MIDAS, Migraine Disability Assessment Scale; VAS, Visual Analog Scale.

Notes: mean ± SD, median (IQR), *n* (%); *Pearson Chi-Squared Test; **Independent Samples T-Test; ***Fisher's Exact Test.

When comparing baseline electrocardiographic parameters between groups, migraine patients had a significantly higher PR interval ( *p* = 0.003). In terms of baseline echocardiographic parameters, migraine patients exhibited higher left ventricular end-systolic diameter (LVESD) ( *p* = 0.024), significantly lower left atrial (LA) diameter ( *p* = 0.006), and lower mitral E wave measurements ( *p* = 0.042) compared with the control group ( Table 2 ).

**Table 2:** Electrocardiographic and echocardiographic parameters of patient and control groups

Parameter	Control ( *n* = 51)	Migraine Patient ( *n* = 50)	*p-value*	Reference range
HR (beats/min)	72.0 (67.0–81.0)	72.5 (70.0–79.7)	0.460 ****	60–100 bpm
PR Interval (ms)	130.0 (118.0–146.0)	146.0 (130.0–160.0)	0.003 ****	120–200 milliseconds
QRS duration (ms)	78.0 (74.0–82.0)	80.0 (75.0–84.0)	0.743 ****	≤ 120 milliseconds
QTc interval (ms)	414.18 ± 25.58	411.73 ± 21.95	0.612 **	< 450 milliseconds (men), < 470 milliseconds (women)
EF (%)	> 50.00	> 50.00	1.000	≥ 50%
LVEDD (mm)	43.0 (40.0–47.0)	44.0 (40.0–47.0)	0.484 ****	42–59 mm (men), 39–53 mm (women)
LVESD (mm)	23.67 ± 3.54	25.46 ± 4.00	0.024 **	25–40 mm (men), 22–35 mm (women)
LA diameter (mm)	30.38 ± 4.55	27.88 ± 4.11	0.006 **	≤ 40 mm
sPAP (mmHg)	25.0 (25.0–27.0)	25.0 (23.7–28.0)	0.719 ****	< 30 mm Hg
Mitral E (cm/s)	85.87 ± 13.44	79.52 ± 16.17	0.042 **	50–90 cm/s
Mitral A (cm/s)	65.0 (55.5–75.0)	70.0 (59.5–80.0)	0.194 ****	30–80 cm/s

Abbreviations: EF, ejection fraction; HR, heart rate; LVEDD, left ventricular end-diastolic diameter; LVESD, left ventricular end-systolic diameter; LA, left atrium diameter; Mitral E/A, Mitral inflow velocities (E-wave and A-wave); sPAP, systolic pulmonary artery pressure.

Notes: **Independent Samples T-test; ****Mann-Whitney U-test.

According to the 24-hour rhythm Holter results, PVCs were detected in 40.0% ( *n* = 20) of the migraine patients. Among these, 38.0% ( *n* = 19) had PVCs constituting < 1% of total heartbeats, while 2.0% ( *n* = 1) had PVCs > 1%. There were no statistically significant differences in the prevalence of PVCs between the migraine and control groups. Supraventricular ectopy was observed in 86.0% ( *n* = 43) of migraine patients. Of these, 82.0% ( *n* = 41) had SVE constituting < 1% of total heartbeats, while 2 patients had SVE > 1%. There were no statistically significant differences in the prevalence of SVEs between the migraine and control groups.

The occurrence of sinus tachycardia was significantly higher in the migraine group, with 39.5% ( *n* = 15) of patients affected, compared with the control group ( *p* = 0.002) ( Table 3 ).

**Table 3:** 24-hour rhythm Holter data of patient and control groups

Parameter		Control ( *n* = 51)	Migraine Patient ( *n* = 50)	*p-value*
Maximum HR (beats/min)		126.63 ± 15.45	128.86 ± 15.67	0.473 **
Mean HR (beats/min)		79.0 (75.0–85.0)	79.5 (73.7–85.0)	0.713 ****
Minimum HR (beats/min)		57.0 (51.0–61.0)	55.5 (50.0–59.2)	0.362 ****
PVC Count		0.0 (0.0–6.0)	0.0 (0.0–5.2)	0.872 ****
Patients with PVCs, *n* (%)		20 (39.2)	20 (40.0)	0.936 *
PVC distribution, *n* (%)	0%	31 (60.8)	30 (60.0)	
	< 1%	19 (37.3)	19 (38.0)	
	> 1%	1 (2.0)	1 (2.0)	
SVE Count		15.0 (1.0–75.0)	15.5 (5.7–93.7)	0.491 ****
Patients with SVE, n (%)		39 (76.5)	43 (86.0)	0.220 *
SVE distribution, *n* (%)	0%	12 (23.5)	7 (14.0)	
	< 1%	39 (76.5)	41 (82.0)	
	> 1%	0	2 (4.0)	
Sinus Tachycardia, n (%)		6 (11.8)	15 (39.5)	0.002 *

Abbreviations: HR, heart rate; PVC, premature ventricular contraction; SVE, supraventricular ectopy.

Notes: *Pearson Chi-squared test; **Independent Samples T-test; ****Mann-Whitney U-test.

The frequency of PVCs was numerically higher in patients with MWA than in those with MWoA; however, this difference was not statistically significant ( *p* > 0.05). Similarly, SVE was less common in MWA than in MWoA, but this difference lacked statistical significance ( *p* > 0.05).

The distribution of data according to the presence of PVCs in control and migraine patients is presented in Table 4 . A significant difference in SVE presence was observed between groups ( *p* = 0.031), driven by a higher frequency of SVEs in migraine patients with PVCs compared with both the control group and migraine patients without PVCs. Similarly, there was a significant difference in the distribution of SVE frequency categories between groups ( *p* = 0.012), attributable to a lower proportion of patients with zero SVEs in the migraine group with PVCs. The distribution of sinus tachycardia also differed among the groups; the occurrence of sinus tachycardia episodes was lower in the control group compared with both migraine subgroups, regardless of PVC status.

**Table 4:** Data distribution in migraine patients based on the presence of PVC

Parameter	Control ( *n* = 51)	No PVC ( *n* = 30)	PVC present ( *n* = 20)	*p-value*
Gender (female), n (%)	39 (76.5)	28 (93.3)	17 (85.0)	0.143 *
Age (years old)	35.0 (28.0–44.0)	42.5 (28.5–46.5)	40.5 (27.0–46.0)	0.316 **
DM, *n* (%)	1 (2.0)	2 (6.7)	3 (15.0)	0.083 ***
HT, *n* (%)	5 (9.8)	2 (6.7)	5 (25.0)	0.152 ***
Comorbidities, *n* (%)	8 (15.7)	4 (13.3)	3 (15.0)	1.000 ***
SVE Count	15.0 (1.0–75.0)	10.5 (0.7–55.2)	50.0 (11.2–135.0)	0.088 **
Patients with SVE, *n* (%)	39 (76.5)	23 (76.7)	20 (100.0)	0.031 ***
SVE Distribution, n (%)				0.012 ***
0%	12 (23.5)	7 (23.3)	0	
< 1%	39 (76.5)	21 (70.0)	20 (100.0)	
> 1%	0	2 (6.7)	0	
HIT-6	–	44.0 (40.0–52.2)	48.5 (42.0–60.7)	0.195 **
Sinus tachycardia, *n* (%)	6 (11.8)	9 (37.5)	6 (42.9)	0.009 *
MIDAS	–	9.0 (6.7–12.0)	13.0 (8.0–21.5)	0.066 ****
VAS	–	7.0 (6.0–8.0)	7.5 (7.0–8.0)	0.223 ****
Photophobia-phonophobia, *n* (%)	–	20 (66.7)	16 (80.0)	0.304 ****

Abbreviations: DM, diabetes mellitus; HT, hypertension; HIT-6, Headache Impact Test-6; MIDAS, Migraine Disability Assessment Scale; PVC, premature ventricular contraction; SVE, supraventricular ectopy; VAS, Visual Analog Scale.

Notes: *Pearson Chi-squared test; **Kruskal-Wallis test; ***Fisher-Freeman-Halton test; ****Mann Whitney U-test.

The Spearman correlation analysis between the rhythm Holter findings and the HIT-6, MIDAS, and VAS scores in migraine patients is summarized in Table 5 . A weak to moderate positive correlation was observed between the HIT-6 score and both the duration of diagnosis (r = 0.316; *p* = 0.025) and the minimum heart rate (Min HR) (r = 0.325; *p* = 0.021). Similarly, a weak to moderate positive correlation was identified between the MIDAS score and the PVC count (r = 0.321; *p* = 0.023). Additionally, the VAS score showed a weak to moderate positive correlation with heart rate and Min HR (r = 0.297; *p* = 0.040; r = 0.311; *p* = 0.028).

**Table 5:** Correlation of HIT-6, MIDAS, and VAS scores with rhythm Holter findings in migraine patients

Parameter	HIT-6	MIDAS	VAS
**Diagnosis duration (months)**	*p* = 0.025** r = 0.316*	*p* = 0.538 r = 0.089	*p* = 0.596 r = 0.077
**HR (bpm)**	*p* = 0.150 r = 0.211	*p* = 0.742 r = 0.049	*p* = 0.040** r = 0.297
**Maximum HR**	*p* = 0.340 r = 0.138	*p* = 0.680 r = 0.060	*p* = 0.738 r = 0.048
**Mean HR**	*p* = 0.197 r = 0.186	*p* = 0.409 r = 0.119	*p* = 0.091 r = 0.242
**Minimum HR**	*p* = 0.021** r = 0.325*	*p* = 0.244 r = 0.168	*p* = 0.028** r = 0.311*
**PVC**	*p* = 0.100 r = 0.235	*p* = 0.023* r = 0.321*	*p* = 0.110 r = 0.229
**SVE**	*p* = 0.381 r = 0.126	*p* = 0.736 r = 0.049	*p* = 0.742 r = 0.048

Abbreviations: HR, heart rate; PVC, premature ventricular contraction; r, spearman correlation coefficients; SVE, supraventricular ectopy.

The present study did not aim to compare the attack and nonattack periods of migraines. However, one patient in the study group experienced a migraine attack while being monitored with a rhythm Holter and underwent repeat data collection during the nonattack period. This patient exhibited a 10-fold increase in PVCs during the migraine attack compared with the attack-free period.

## DISCUSSION

The present study demonstrated that migraine patients have a higher prevalence of sinus tachycardia and prolonged PR intervals during pain-free intervals, with no significant increase in PVC frequency.

Migraine is a chronic and often lifelong condition that affects > 1 billion individuals globally, highlighting its substantial global burden and the need to understand its associated cardiovascular risks. 17 It is associated with an increased risk of ischemic stroke and ischemic heart disease, particularly among women and individuals experiencing MWA. 18 19 Migraine patients also exhibit higher rates of hypertension and hypercholesterolemia, further contributing to their elevated cardiovascular risk. 1 The 2021 guidelines from the European Society of Cardiology (ESC) on cardiovascular disease prevention recommend including MWA as a factor in cardiovascular risk assessment. 20 Migraine can also influence the autonomic nervous system, which may lead to cardiac arrhythmias. Recent studies suggest that patients with MWA have an increased risk of AF, potentially mediated by autonomic dysfunction and vascular risk factors, both of which are more pronounced in MWA patients compared with MWoA patients. 21

Premature ventricular contractions, defined as early beats originating from ectopic ventricular foci, may arise from mechanisms such as re-entry due to scar tissue, triggered activity from delayed afterdepolarizations, or enhanced automaticity caused by increased catecholamine release. 22 These mechanisms might be influenced by autonomic dysfunction in migraine patients, potentially linking migraine-associated cardiovascular alterations with arrhythmogenesis. While the relationship between ventricular arrhythmias and migraine has been explored in a limited number of studies, our study specifically investigated the association between PVCs and the attack-free period in migraine patients, addressing an important gap in the current literature.

Our results showed no significant differences in QRS duration, QTc, or mean heart rate on electrocardiography between migraine patients during the nonattack period and healthy individuals. Likewise, the frequency of PVCs on 24-hour rhythm Holter monitoring was similar between the 2 groups. These findings suggest that autonomic disturbances affecting ventricular condition during migraine attacks may normalize during the nonattack period. 7 This is consistent with the well-established influence of autonomic conditions on the ventricular myocardium, which can lead to variations in QT intervals independent of heart rate. 23 A meta-analysis has shown that migraineurs are prone to longer QTcs and a higher likelihood of QTc prolongation during migraine attacks compared with pain-free periods. 24 Our study further supports that, during the migraine-free period, PVC frequency and QT intervals are similar to those observed in healthy individuals, indicating reversibility of autonomic disturbances. Additionally, we found no significant differences in the number or frequency of PVCs and SVE during pain-free periods between MWA and MWoA patients. Although the number of MWA patients in our study was limited, this subgroup is theoretically at higher cardiovascular risk. Future research including larger cohorts of MWA patients is warranted to clarify these associations.

Several studies have reported no significant difference in PR intervals between the healthy population and migraine patients. 13 25 26 However, in our study, the PR interval was significantly longer in migraine patients compared with the control group, although both values remained within the normal range. Aygun et al. previously demonstrated that PR intervals differ significantly between pain-free periods and migraine attacks. 7 Additionally, a meta-analysis identified PR interval prolongation as an independent risk factor for AF incidence. 27 Our findings are in line with these observations, providing further insight into the potential relationship between migraine and atrial arrhythmias.

Our results indicate that the frequency of sinus tachycardia during the day was significantly higher in the migraine group compared with the control group. This finding suggests that heightened sympathetic stimulation may persist even during nonattack periods, potentially contributing to an increased cardiovascular risk. Although the frequency of sinus tachycardia in the migraine group (39.5%) appears higher than expected, direct comparison with normative 24-hour Holter data is limited due to variability in definitions and populations. A formal measure of effect size, such as odds ratio, was not calculated in the present study, but would provide a more precise estimate of the strength of association in future research. However, these results are in line with previous studies linking migraine to sympathetic dysfunction. 7 10 Moreover, autonomic nervous system dysfunction in migraine patients could play a role in the long-term elevation of cardiovascular risk. 28 A similar mechanism has also been proposed to explain the higher prevalence of AF and ventricular arrhythmias observed in migraine patients. 29 These findings emphasize the need for further research to evaluate whether closer cardiovascular monitoring may be warranted in this population.

The Headache Impact Test and MIDAS are widely used patient-reported outcome measures (PROMs) validated for assessing migraine severity and treatment effectiveness. Additionally, the visual analog scale (VAS) is a reliable tool frequently employed to evaluate treatment outcomes in migraine patients. 30 31 32 In our study, we observed a low-to-moderate correlation between PVC frequency and MIDAS scores in the migraine group, suggesting a potential link between migraine burden and cardiac arrhythmias. This finding may highlight the need for closer cardiovascular monitoring in patients with high migraine-related disability, as reflected in their MIDAS scores. However, given the complex pathophysiology of migraine and the multifactorial nature of PVC occurrence, as well as possible confounding variables and the risk of type I error, this finding should be interpreted with caution. Further studies are necessary to confirm and expand on these findings, as well as to elucidate the underlying mechanisms.

One of the primary limitations of the present study is the small sample size, restricting the generalizability of our findings. The relatively short duration of migraine diagnosis in patients also limited the assessment of long-term effects. The predominance of MWoA in the study group further limited our ability to thoroughly analyze the specific effects in the subgroup of patients with MWA. Approximately 18% of patients were using tricyclic antidepressants, which may influence cardiac conduction parameters. However, given their common use in migraine prophylaxis, this reflects a real-world population and was not an exclusion criterion. The absence of a predefined time interval between the last migraine attack and Holter monitoring may have affected the recorded outcomes. We did not evaluate or adjust for anxiety disorders, which are common comorbidities of migraine and may have influenced the presence of sinus tachycardia. Finally, the single-center design of the study constrained the assessment of potential variations across populations with different geographic or demographic characteristics.

In conclusion, the present study is the first cross-sectional investigation, to our knowledge, comparing PVC frequency in migraine patients during the attack-free period with healthy individuals. Our findings indicate that migraine patients do not show a significant difference in PVC prevalence compared with healthy controls during the nonattack period. Additionally, a higher frequency of sinus tachycardia was found in migraine patients, which may reflect persistent autonomic dysregulation. Despite these findings, structural heart abnormalities were not identified, suggesting that autonomic imbalance may be a primary factor influencing arrhythmias in migraine patients. Overall, our study highlights the need for further research with larger populations to clarify these relationships and explore the long-term cardiovascular implications of migraine.
